# Cytotoxic Cycloartane Triterpenoid Saponins from the Rhizomes of *Cimicifuga foetida*

**DOI:** 10.1007/s13659-019-0214-1

**Published:** 2019-06-18

**Authors:** Jing Lu, Xing-Rong Peng, Da-Shan Li, Qiang-Qiang Shi, Ming-Hua Qiu

**Affiliations:** 1 State Key Laboratory of Phytochemistry and Plant Resources in West China, Kunming Institute of Botany, Chinese Academy of Sciences (CAS), Kunming, 650204 People’s Republic of China; 2 University of Chinese Academy of Sciences, Beijing, 100049 China; 3 State Key Laboratory of Phytochemistry and Plant Resources in West China, Kunming Institute of Botany, Chinese Academy of Sciences, 132 LanHei Road, Kunming, 650201 Yunnan People’s Republic of China

**Keywords:** *Cimicifuga foetida*, Cycloartane triterpenoid saponins, Cytotoxic activity

## Abstract

**Abstract:**

To enrich the bioactive cycloartane triterpenoid glycoside named actein and find out more cytotoxic cycloartane triterpenes, a phytochemical study of *Cimicifuga foetida* was conducted. 113 g (0.17%) actein was purified by recrystallization while eight cycloartane-type triterpenes (**1**–**8**) were isolated from the mother liquid. The chemical structures of new compounds (**1**–**4**) were elucidated by 1D and 2D NMR and HRESIMS spectroscopic analyses. Moreover, new compounds showed moderate and broad-spectrum cytotoxicity against 5 human cancer cell lines with IC_50_ values ranging from 4.02 to 15.80 *μ*M.

**Graphic Abstract:**

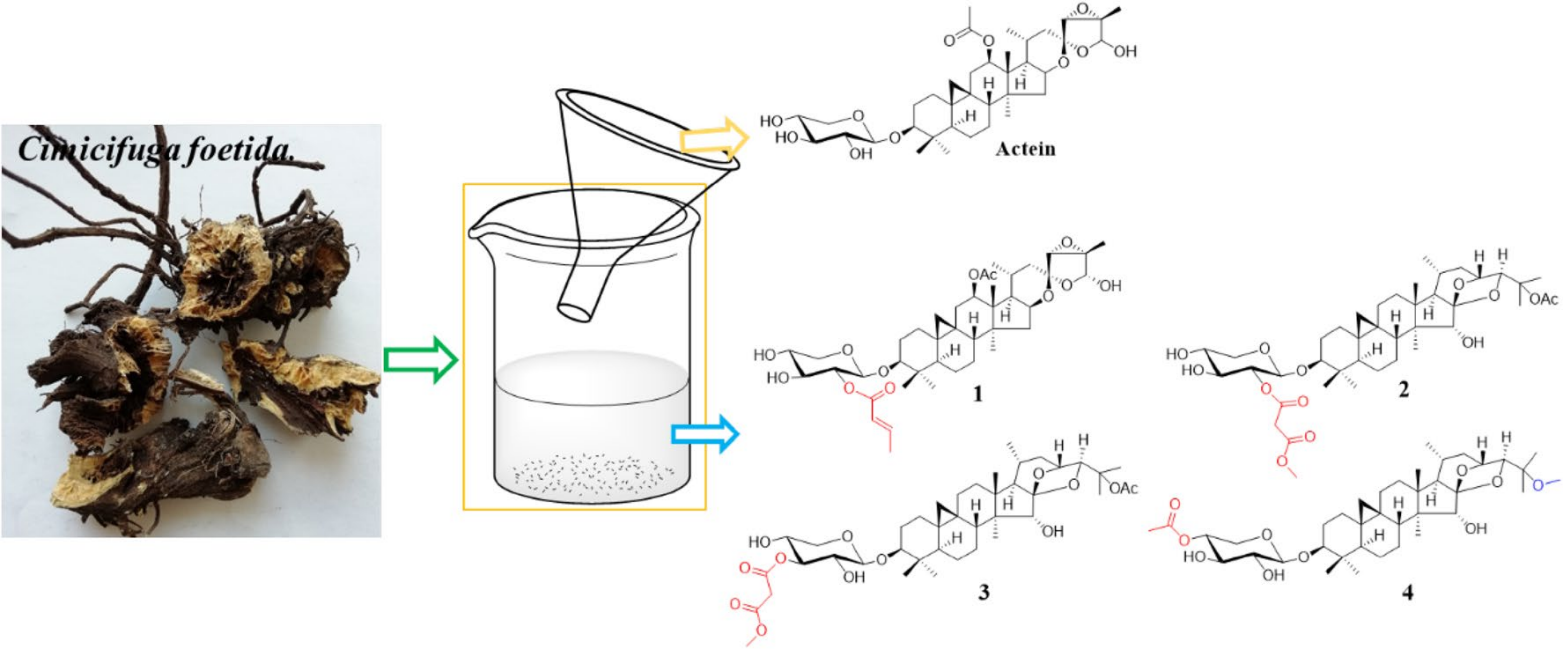

**Electronic supplementary material:**

The online version of this article (10.1007/s13659-019-0214-1) contains supplementary material, which is available to authorized users.

## Introduction

*Cimicifuga foetida,* commonly known as “shengma”, is a famous traditional chinese medicine native to china. The roots of *C. foetida* have been widely used to relieve fever and inflammation for thousand years and also have been officially listed in the Chinese Pharmacopoeia [1, 2]. During the past decades, the cyclolanostane triterpenoid glycosides of *Cimicifuga* genus have been noticed for their strong cytotoxic activities on cancer, especially breast cancer. Actein is the representative cyclolanostane triterpene isolated from *Cimicifuga*, exhibited an anti-angiogenic and anti-cancer activities on human breast cancer models by suppressing the protein expressions JNK/ERK pathways. Further anti-metastatic activity study showed that actein could down-regulated the protein expression of epidermal growth factor receptor (EGFR) and affect AKT and NF-κB pathways [3–5]. In addition, actein also showed cytotoxic activities against the hepatobiliary cancer, glioma growth, liver cancer and cervical cancer [6–12]. Because the anti-breast cancer activity of actein, the pharmacodynamics, pharmacology and toxicology experiments were tested by us. In order to carry out the preclinical research of actein, a simply and fast method is needed to enrich considerable amount of actein from the roots of *C. foetida*.

Massive experiments found that triterpenoid saponins which have the similar polar with actein show similar cytotoxic effects [13–15]. Meanwhile, analysis of the mother liquid after recrystallization by TLC (thin-layer chromatography) and HPLC (high performance liquid chromatography) showed that a small amount of cycloartane triterpenes still existed. In order to further explore the bioactivities metabolites from *C. foetida*, the mother liquid was selected for systematically investigated and four new compounds (**1**–**4**), acteol-3-*O*-[2′-*O*-(*E*)-2-butenoyl]-*β*-d-xylopyranoside (**1**), 25-*O*-acetylcimigenol-3-*O*-[2′-*O*-3-methoxy-3-oxo-propionyl]-*β*-d-xylopyranoside (**2**), 25-*O*-acetylcimigenol-3-*O*-[3′-*O*-3-methoxy-3-oxo-propionyl]-*β*-d-xylopyranoside (**3**), 25-*O*-methoxycimigenol-3-*O*-[4′-*O*-acetyl]-*β*-d-xylopyranoside (**4**), and four known ones 25-*O*-acetylcimigenol-3-*O*-[2′-*O*-(*E*)-butenoyl]- *α*-l-arabinopyranoside (**5**), Soulieoside A (**6**), 3′-*O*-acetylactein (**7**), 25-*O*-acetycimigenol-3-*O*-*β*-d-xylopyranoside (**8**) were isolated. New compounds (**1**–**4**) were evaluated for their cytotoxic activities against human HepG2, SMMC-7721, A549, MCF-7, and SW-480 cancer cell lines by using the MTT assay. Herein, the recrystallization process of actein, the isolation, structure’s elucidation, and biological activities of new isolates are described.

## Results and Discussion

Actein obtained by simply repeated recrystallization. And eight triterpenoid saponins (**1**–**8**) were isolated from the mother liquid. The structure elucidation of new compounds (**1**–**4**) are as followed.

Actein was obtained as white needles. The details of recrystallization process can be found in experiments section.

Compound **1** was obtained as white, amorphous powder. And the molecular formula C_41_H_60_O_12_ was deduced from its HRESIMS data, with a deprotonated molecular ion [M−H]^−^ at *m/z* 743.4011 (calcd 743.4012). IR absorption bands at 3436 and 1734 cm^−1^ indicated the presence of hydroxy and carbonyl groups. The ^1^H NMR spectrum of **1** showed the resonances of nonequivalent protons of a cyclopropyl methylene signal [*δ*_H_ 0.18 and 0.51 (each 1H, d, *J *= 3.6 Hz, H-19a/H-19b)], a secondary [*δ*_H_ 0.94 (d, *J *= 6.2 Hz, Me-21)], and five tertiary methyl groups [*δ*_H_ 1.34 (s, Me-18), 1.77 (s, Me-27), 0.76 (s, Me-28), 1.11 (s, Me-29), and 0.94 (s, Me-30)], an acetyl group [*δ*_H_ 2.13 (s, -OAc)], two olefinic protons [*δ*_H_ 6.10 (1H, d, *J *= 15.6 Hz) and 7.15 (1H, m)], an anomeric proton [*δ*_H_ 4.88 (d, *J *= 7.9 Hz, H-1′)]. The above evidence, together with the characteristic signals for a ketal carbon [*δ*_C_ 105.7 (C-23)] in the ^13^C NMR spectrum, confirmed that **1** is a 9,19-cimigenol-type monoglycoside with one acetoxy group.

**Fig. 1:**
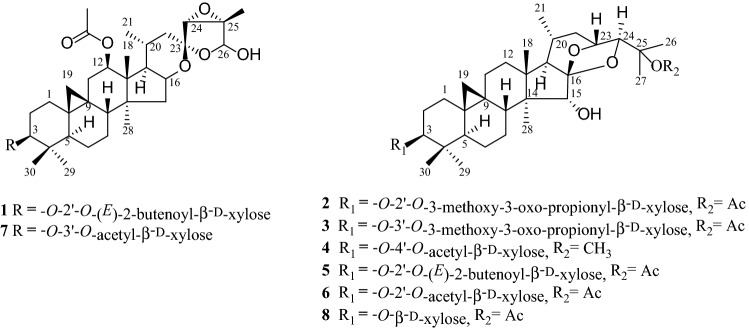
Structures of compounds **1**–**8**

**Fig. 2:**
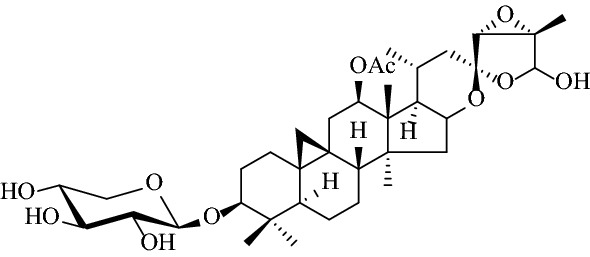
The structure of actein

**Fig. 3:**
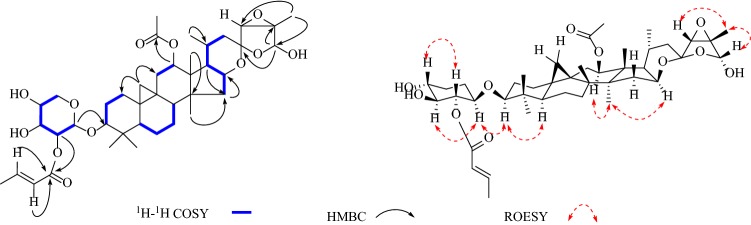
Key correlations in 2D NMR spectra of compound **1**

In addition, the diagnostic HMBC correlations observed from [*δ*_H_ 5.72 (s, H-26)] to two quaternary carbon resonances [*δ*_C_ 105.7 (C-23) and 65.4 (C-25)], and [*δ*_H_ 1.77 (s, Me-27)] to a quaternary carbon resonance at [*δ*_C_ 65.4 (C-25)] and two methine carbon resonances at [*δ*_C_ 98.3 (C-26) and 63.3 (C-24)] indicated that **1** was acteol-type triterpenoid. In the ^13^C NMR (DEPT) spectra, the signals ascribable to an α, *β*-unsaturated ketone moiety at *δ*_C_ 165.9 (s), 122.1 (d), 149.7 (d), 31.6 (q) were observed. A comparison of the spectroscopic data of **1** with actein [16] showed that, structurally, **1** closely resembles actein, with the main differences of the sugar moiety, including the *α,β*-unsaturated ketone resonances. And in the ^1^H-^1^H COSY spectrum (Fig. 3), a correlation was observed between the methyl signal [*δ*_H_ 2.05 (d, *J* = 6.2 Hz, H-4′′)]and the olefinic proton [*δ*_H_ 7.15 (m, H-3′′)], which indicated the tetra-carbon unit to be a 2-butenoyl group. In addition, the coupling constant (*J* = 15.6 Hz) of the two olefinic protons [*δ*_H_ 6.10 (H-2′′) and 7.15 (H-3′′)] confirmed the *E*-geometry of a double bond in the 2-butenoyl moiety. Moreover, the observed HMBC correlation of [*δ*_H_ 5.65 (m, H-2′)] with the carbonyl of 2-butenoyl suggested that 2-butenoyl was connected to [*δ*_C_ 75.3 (C-2′)]. Meanwhile, [*δ*_H_ 5.07 (m, H-12)] shows HMBC correlation with an ester carbonyl carbon at [*δ*_C_ 170.5 (-OAc)], therefore, a acetyl group is located at C-12. Finally, in the HMBC spectrum (Fig. 1), the correlation of the anomeric proton [*δ*_H_ 4.88 (d, *J *= 7.9 Hz, H-1′)] with the methine carbon [*δ*_C_ 88.2 (C-3)] illustrated that the sugar moiety was located at C-3. After acid hydrolysis, the sugar was identified as D-xylose by comparing its TLC and specific rotation with an authentic sample (Fig. 2).

In the ROESY spectrum (Fig. 3), the d-xylose moiety was suggested to be in the *β*-configuration by the large coupling constant of the anomeric proton (*J*_1,2_ = 7.9 Hz) in the ^1^H NMR spectrum. Additionally, when combined with the key correlations observed in the ROESY spectrum of H-1′ (axial), H-2′ (equatorial), H-3′ (axial), and H-4′ (equatorial), it indicated that C-2′, C-3′, and C-4′ are *α*-, *β*-, and *α*-configurations, respectively. These findings confirmed that the monoglycoside unit of **1** is a xylopyranoside moiety. Moreover, correlations between H-3/H-5, H-12/H-28, H-16/H-28 suggested *α*-orientation of H-3, H-12 and H-16.The correlations between H-24/H-27 and H-26/H-27 in ROESY spectrum suggested *β*-orientation of H-24, Me-27 and H-26 and indicated that compound **1** is stable of *α*-OH (C-26). The absolute configuration of C-23 was established by comparing to the ^13^C NMR spectroscopic data of 23-*epi*-26-deoxyActein (23*S*) and actein (23*R*). When the chemical shifts of C-16 and C-20 were *δ*_C_ 73.0 and 26.0, the absolute configuration of C-23 was assigned as *R*, similar to actein, while signals at *δ*_C_ 74.5 and *δ*_C_ 23.3 were corresponding to *S*-configuration, similar to 23-*epi*-26-deoxyActein [17]. Thus, chemical shifts of C-16 and C-20 were *δ*_C_ 25.9 and 72.9 in compound **1**, respectively, indicating that the absolute configuration of C-23 was *R.* Therefore, the structure of **1** was elucidated as acteol-3-*O*-2′-*O*-[(*E*)-2-butenoyl]-*β*-d-xylopyranoside.

Compound **2** was obtained as a white, amorphous powder, showing an [M−H]^−^ ion at *m/z* 761.4119 in the HR-ESIMS consistent with the empirical molecular formula C_41_H_62_O_13_ (calc. 761.4118), requiring 11 sites of unsaturation. The IR spectrum showed absorptions of hydroxy and carbonyl groups at 3436 and 1739 cm^−1^, respectively. In the ^13^C NMR (DEPT) spectrum, 41 carbon signals could be resolved as nine methyls, ten methylenes, twelve methines, and ten quaternary carbons. In the ^1^H NMR spectrum of **2**, the characteristic cyclopropane methylene resonances at *δ*_H_ 0.23 and 0.46 (each 1H, d, *J *= 3.8 Hz), an anomeric proton at *δ*_H_ 4.81 (1H, d, *J *=7.9 Hz), an acetyl methyl at *δ*_H_ 1.66 (1H, s, -OAc), a secondary methyl resonances at *δ*_H_ 0.83 (1H, d, *J *=6.4 Hz), and seven tertiary methyl groups [*δ*_H_ 1.10 (3H, s, Me-18), *δ*_H_ 1.66 (3H, s, Me-26), *δ*_H_ 1.64 (3H, s, Me-27), *δ*_H_ 1.17 (3H, s, Me-28), *δ*_H_ 1.09 (3H, s, Me-29), *δ*_H_ 0.98 (3H, s, Me-30) and *δ*_H_ 3.63 [(3H, s, Me-4′′)] were observed. The above evidences, together with the diagnostic signals for two oxygen-bearing methine carbons [*δ*_C_ 71.5 (C-23) and 86.6 (C-24)] and a ketal carbon [*δ*_C_ 112.3 (C-16)] in the ^13^C NMR spectrum, confirmed that **2** is a 9,19-cimigenol-type monoglycoside with one acetoxy group. The ^13^C NMR spectrum also revealed carbons assignable to a 3-methoxy-3-oxo-propionyl moiety [*δ*_C_ 167.0 (s, C-1′′), 41.8 (t, C-2′′), 166.3 (s, C-3′′) and 52.1 (q, C-4′′)] and a glycosidic moiety [*δ*_C_ 104.0 (d, C-1′), 75.9 (d, C-2′), 76.6 (d, C-3′), 71.1 (d, C-4′), and 66.9 (t, C-5′)].

**Fig. 4:**
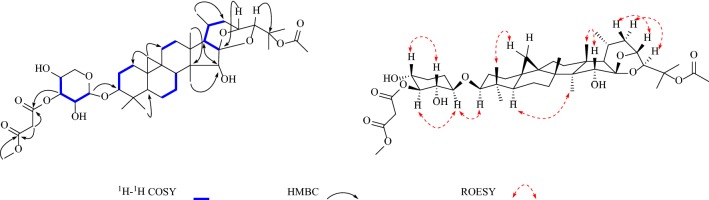
Key correlations in 2D NMR spectra of compound **3**

In the HMBC spectrum (Fig. 4), the correlation of the proton at *δ*_H_ 4.81 (d, *J *= 7.9 Hz, H-1′) with the methine carbon at *δ*_C_ 88.5 (d, C-3) indicated that the sugar moiety was located at C-3. Aforementioned data indicated that the structure of **2** was similar to 25-*O*-acetylcimigenol-3-*O*-*β*-d-xylopyranoside (**8**) [18], with the only difference in the sugar moiety. In **8** the H-2′ resonance was observed at *δ*_H_ 4.02, whereas in **2** it shifted downfield to *δ*_H_ 5.52. In addition, the C-1′ resonances at *δ*_C_ 107.5 and C-3′ at *δ*_C_ 78.6 in **8** shifted upfield to *δ*_C_ 104.0 and 76.6, respectively, in **2**. Which may be explained by the presence of the C-2′ 3-methoxyl-3-oxo-propionyl moiety group of the xylose unit. This deduction was confirmed by the HMBC correlation observed between the correlation of the proton at *δ*_H_ 5.52 (1H, t, *J *= 8.6 Hz, H-2′) with the ester carbonyl carbon at *δ*_C_ 167.0 (s, C-1′′).

In the ROESY spectra (Fig. 4), the d-xylose moiety was suggested to be in the *β*-configuration by the large coupling constant of the anomeric proton (*J*_1,2_ = 7.9 Hz) in the ^1^H NMR spectrum. Moreover, correlations of H-3/H-1′, H-3/H-5, H-15/H-18, H-23/H-22a, H-24/H-22b suggested *α*-orientation at H-3, H-23, *β*-orientation at H-24 and H-15. The absolute configuration of C-24 was assigned as *s* because the chemical shifts of C-24 in **2** resembles those of 24*S*-compound [*δ*_C_ 86.47 and *δ*_H_ 4.13 (1H, s)] instead of 24*R*-compound [*δ*_C_ 84.1 and *δ*_H_ 3.77 (1H, d, *J *= 4.4 Hz)] [19]. The configuration at C-23, C-24 were assigned as *R* and *S*, respectively. Therefore, the structure of **2** was elucidated as 25-*O*-acetylcimigenol-3-*O*-[2′-*O*-3-methoxy-3-oxo-propionyl]-*β*-d-xylopyranoside.

Compound **3** gave the same molecular formula as compound **2** by HR-ESIMS. The IR and 1D NMR spectra of compound **3** showed close resemblances to those of **2** except for slight differences resonances of the sugar moiety. In **2**, the H-3′ resonance was observed at *δ*_H_ 4.12 m, whereas in **3** it shifted downfield to *δ*_H_ 5.73 (1H, t, *J *= 9.2 Hz). In addition, the C-2′ resonances at *δ*_C_ 75.9 and C-4′ at *δ*_C_ 71.1 in **2** shifted upfield to *δ*_C_ 72.8 and 68.9, respectively, in **3**, which may be explained by the presence of the C-3′ 3-methoxy-3-oxo-propionyl moiety of the xylose unit. Which was further proved by the HMBC correlation. And used the same method as compound **2**, the configuration at C-23, C-24 were assigned as *R* and *S*, respectively. Thus, compound **3** was determined as 25-*O*-acetylcimigenol-3-*O*-[3′-*O*-3-methoxy-3-oxo-propionyl]-*β*-d-xylopyranoside.

Compound **4** gave a molecular formula of C_38_H_60_O_10_ by HR-ESIMS, and the ^1^H NMR spectra displayed characteristic cycloartane proton signals, including a cyclopropane methylene *δ*_H_ 0.27 and 0.51 (each 1H, d, *J* = 3.6 Hz, H-19), and six methyl proton signals at *δ*_H_ 1.02, 1.12, 1.17, 1.24, 1.26, 1.30 (each 3H, s), secondary methyl resonances at *δ*_H_ 0.83 (3H, d, *J* = 6.4 Hz) and methoxy group at *δ*_H_ 3.18 (3H, s). An *β*-d-xylopyranoside unit was observed by comparison of the 1D NMR data with those of compounds **2 **− **3** (Tables 1, 2). The 1D NMR spectra (Tables 1, 2) showed **4** were similar to 4′-*O*-acetylcimigenol-3-*O*-*β*-d-xylopyranoside [20], except for the presence of an additional methoxy group. Furthermore, the position of the attached methoxy group was supported by a downfield chemical shift of *δ*_C_ 76.2 (C-25) in the ^13^C NMR spectra (Table 2) and HMBC correlations of *δ*_H_ 3.18 (OMe) with C-25 (*δ*_C_ 76.2), and between *δ*_H_ 1.30 (s, CH_3_-26) and 1.24 (s, CH_3_-27) with C-25 (*δ*_C_ 76.2). From these datas, it was concluded that there is a methoxy group attachment at C-25 of compound **4**.

**Table 1:** ^1^H (600 MHz) NMR data of compounds **1**–**4** in Pyridine-d_5_ [*δ* in ppm, *J* in Hz]

No.	**1**	**2**	**3**	**4**
1	1.17 m, 1.56 m	1.18^a^, 1.52 m	1.21^a^, 1.54 m	1.24^a^, 1.54 m
2	1.97 m, 2.20 m	1.98 s, 2.28 m	1.94 m, 2.29 m	1.94 m, 2.29 m
3	3.35 dd (4.8, 11.5)	3.37 dd (4.3, 11.6)	3.48 dd (4.2, 11.5)	3.48 dd (4.2, 11.7)
5	1.19 m	1.29^a^	1.30^a^	1.17^a^
6	0.81 m, 1.41 s	0.70 m 1.51^a^	0.70 m 1.52^a^	0.70 m 1.53^a^
7	0.88^a^, 1.02^a^	1.08^a^, 2.07 m	1.07^a^, 2.08 m	1.07^a^, 2.08^a^
8	1.53^a^	1.64^a^	1.65^a^	1.65^a^
11	1.15 m, 2.69 m	1.18^a^, 2.12 m	1.18^a^, 2.10 m	1.18^a^, 2.10 m
12	5.07 m	1.52 m, 1.63^a^	1.51 m, 1.65^a^	1.51 m, 1.65^a^
15	1.51^a^, 1.72^a^	4.24 s	4.25 s	4.25 s
16	4.59 m			
17	1.76 m	1.42 m	1.44 m	1.44 m
18	1.34 s	1.10 s	1.13 s	1.12 s
19	0.51 d (3.6) 0.18 d (3.6)	0.46 d (3.8) 0.23 d (3.8)	0.51 d (3.8) 0.28 d (3.8)	0.51 d (3.6) 0.27 d (3.6)
20	1.80^a^	1.66^a^	1.67^a^	1.65^a^
21	0.94 d (6.4)	0.83 d (6.4)	0.83 d (6.4)	0.83 d (6.4)
22	1.63^a^, 2.21 m	0.94^a^ 2.21 m	0.96^a^ 2.24 m	0.96 m 2.29 m
23		4.57 d (9.1)	4.58 d (9.0)	4.60 d (9.0)
24	3.92 s	4.09 s	4.02 m	4.03 m
25				
26	5.72 s	1.66 s	1.67 s	1.30 s
27	1.77 s	1.64 s	1.65 s	1.24 s
28	0.76 s	1.17 s	1.17 s	1.17 s
29	1.11 s	1.09 s	1.10 s	1.26 s
30	0.94 s	0.98 s	1.01 s	1.02 s
AcO-12	2.13 s			
AcO-25		1.66 s	1.95 s	
CH_3_O-25				3.18 s
AcO-4′				1.23 s
1′	4.88 d (7.9)	4.81 d (7.9)	4.82 d (7.4)	4.85 d (7.5)
2′	5.65 m	5.52 t (8.6)	4.01 m	4.02 t (7.9)
3′	4.16 m	4.12 m	5.73 t (9.2)	4.26 t (8.9)
4′	4.23 m	4.27 m	4.21 m	5.37 m
5′	3.71 t (10.5) 4.31 dd (11.3, 5.0)	3.63 t (10.9) 4.25 dd (5.2, 11.3)	3.69 t (10.7) 4.31 dd (5.3, 11.3)	3.59 t (11.0) 4.32 dd (5.4, 11.2)
1′′				
2′′	6.10 d (15.6)	3.75 s	3.62 s	
3′′	7.15 m			
4′′	2.05 d (6.2)	3.63 s	3.52 s	

^a^Signals overlapped

**Table 2:** ^13^C (150 MHz) NMR data of compounds **1**–**4** [*δ* in ppm, *J* in Hz]

No.	**1**	**2**	**3**	**4**
1	32.1 t	32.1 t	32.3 t	32.3 t
2	29.6 t	30.7 t	30.7 t	30.7 t
3	88.2 d	88.5 d	88.7 d	88.5 d
4	40.9 s	40.9 s	41.2 s	41.7 s
5	46.7 d	47.4 d	47.4 d	47.4 d
6	20.3 t	20.9 t	20.9 t	20.9 t
7	25.5 t	26.2 t	26.2 t	26.2 t
8	45.7 d	48.5 d	48.5 d	48.5 d
9	19.4 s	19.9 s	19.4 s	19.9 s
10	26.5 s	26.4 s	26.5 s	26.5 s
11	36.6 t	26.3 t	26.3 t	26.3 t
12	76.9 d	33.9 t	33.9 t	33.9 t
13	48.6 s	41.7 s	41.7 s	41.2 s
14	47.6 s	47.0 s	47.0 s	47.1 s
15	43.4 t	80.0 d	80.0 d	80.0 d
16	72.9 d	112.3 s	112.3 s	111.8 s
17	56.3 d	59.3 d	59.3 d	59.3 d
18	13.4 q	19.4 q	19.4 q	19.4 q
19	29.4 t	29.8 t	29.9 t	29.9 t
20	25.9 d	23.8 d	23.8 d	23.9 d
21	20.9 q	19.4 q	19.4 q	19.2 q
22	37.5 t	37.8 t	37.8 t	37.9 t
23	105.7 s	71.5 d	71.5 d	71.5 d
24	63.3 d	86.6 d	86.6 d	88.1 d
25	65.4 s	83.0 s	83.0 s	76.2 s
26	98.3 d	23.3 q	23.3 q	20.8 q
27	13.0 q	21.4 q	21.4 q	19.5 q
28	19.3 q	11.7 q	11.7 q	11.7 q
29	25.4 q	25.4 q	25.5 q	25.6 q
30	15.0 q	15.1 q	15.2 q	15.3 q
AcO-12	170.5 s			
	21.5 q			
AcO-25		170.0 s	170.0 s	
		22.2 q	22.2 q	
CH_3_O-25				49.1 q
AcO-4′				170.5 s
				22.0 q
1′	104.7 d	104.0 d	107.0 d	107.2 d
2′	75.3 d	75.9 d	72.8 d	75.6 d
3′	76.3 d	76.6 d	80.4 d	74.8 d
4′	71.2 d	71.1 d	68.9 d	73.0 d
5′	67.0 t	66.9 t	66.6 t	63.01 t
1′′	165.9 s	167.0 s	167.3 s	
2′′	122.1 d	41.8 t	41.8 t	
3′′	149.7 d	166.3 s	166.9 s	
4′′	31.6 q	52.1 q	52.9 q	

**Fig. 5:**
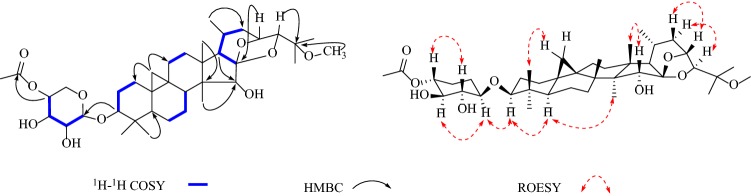
Key correlations in 2D NMR spectra of compound **4**

The ROESY correlations (Fig. 5) of H-3 with H-1′, H-3 with H-5, H-15 with H-28, H-23 with H-22a and H-24 with H-22b illustrated that both H-3 and H-23 were *α*-oriented, H-24 and H-15 were *β*-orientation. And used the same method as compound **2,** the configurations at C-23 and C-24 were assigned as *R* and *S*. Therefore, the structure of **4** was elucidated as 25-*O*-methylcimigenol-3-*O*-[4′-*O*- acetyl]-*β*-d-xylopyranoside.

Four know compounds, soulieoside A (**6**) [21], 25-*O*-acetylcimigenol-3-*O*-[2′-*O*-butenoyl]-*α*-l-arabinopyranoside (**5**) [22], 3′-*O*-acetyl-actein (**7**) [23], 25-*O*-acetylcimigenol-3-*O*-*β*-d-xylopyranoside (**8**), were isolated and identified.

These isolated new compounds (**1**‒**4**) were evaluated for their cytotoxicities against HL-60, SMMC-7721, A-549, MCF-7, and SW-480 cell lines (Table 3). Compounds **1**‒**4** showed significant cytotoxic activities against five human tumor cells with IC_50_ values ranging from 4.02 to 15.80 *μ*M. Especially, all the new compounds exhibited stronger cytotoxicities against A-549, MCF-7 and SW-480 cell lines than SMMC-7721 and HL-60 cell lines, even stronger than positive control (Cisplatin). Based on the above results, we also found that the cytotoxic activities of new compounds, which means these substituents (like acetyl, butenoyl or methoxyl-oxo-propionyl) attached to sugar unit may contribute to inhibition activity.

**Table 3:** IC_50_ values (IC_50_) of compounds **1**–**4** for human tumor cell lines (n = 3)

Compounds	HL-60	A-549	SMMC-7721	MCF-7	SW480
**1**	16.79	7.64	5.86	10.77	14.47
**2**	14.82	9.87	4.02	14.93	14.50
**3**	14.74	14.59	15.76	16.31	14.65
**4**	15.80	14.35	14.21	13.52	13.43
Cisplatin	3.24	14.72	5.00	17.87	21.66

In this present study, a great amount of high-purity actein were obtained by recrystallization, which laid a material foundation for the pre-clinical research. Compounds **1**‒**4** isolated from the mother liquid also showed inhibition activity to different cancer cell lines, which provide not only chemical model for discovering potential anticancer agents, but also a proof for further development and utilization of *Cimiciguga* genus.

## Experiments Section

### General Experimental Procedures

Optical rotation was obtained in methanol with a JASCO P-1020 digital polarimeter (Jasco, Tokyo, Japan). A Shimadzu UV2401PC spectrophotometer (Shimadzu, Kyoto, Japan) was used to obtain ultraviolet (UV) spectra. HREIMS data were measured on a Waters API QSTAR Pulsar spectrometer. HPLC–UV/MS analysis was taken on an Agilent 1290 HPLC/Q-TOF mass system with a ZORBAX SB-C18 column (5 *μ*m, 4.6 mm × 250 mm, 0.8 mL/min). A Bruker Tensor-27 Fourier transform infrared spectrometer (Bruker, German) was used for scanning IR spectra with KBr pellets. 1D and 2D NMR spectra were obtained on Bruker Ascend-600 MHz NMR spectrometers (Bruker, Zurich, Switzerland). Column chromatography (CC) was performed on macroporous resin (D101, Tianjin Haoju Science and Technology Ltd.), Silica gel (200–300 mesh, Qingdao Marine Chemical, Ltd.), Land Sephadex LH-20 (20–150 *μ*m, Pharmacia).

### Plant Material

Rhizomes of *C. foetida* (67 kg) were collected from Yulong County, Yunnan Province, China, in August 2014. The material was identified by Prof. Shengji Pei at Kunming Institute of Botany, Chinese Academy of Science. A Voucher specimen (KUN No. 20140828) has been deposited at the State Key Laboratory of Phytochemistry and Plant Resources in West China, Kunming Institute of Botany, Kunming, China.

### Extraction and Isolation

The air-dried and powdered rhizomes of *C. foetida* (67 kg) were extracted three times with 90% aqueous methanol (50 L × 3) at 60 °C to give a residue after evaporating under vacuum at 50 °C. The residue was suspended in water and then partitioned with petroleum ether (PE), EtOAc and n-BuOH. The EtOAc portion (8 kg) was subjected to the D101 macroporous absorption resin and eluted with a gradient of (MeOH/H_2_O = 50:50, 70:30, 90:10) to afford three fractions. The fraction (MeOH/H_2_O = 90:10, 4 kg) was fragmented by a silica gel column (CHCl_3_/MeOH = 100:1, 50:1, 20:1) and yields three subfractions. Further analysis of TLC and HPLC showed that actein existed in the (CHCl_3_/MeOH = 50:1, 160 g) fraction. A multi-solvent (MeOH/MeCO_2_ = 1:3) recrystallization was used to purify the actein.

The mixture (160 g) was dissolved in the MeOH (300 ml), which was a saturated solution at 45 °C. Then filter the solution to remove the insoluble impurities. Later, MeCO_2_ (600 ml) was added to above solution to obtain a mixed solvent system. The mixed solvent system allowed to cool over time to give crystals. The next step, separated the crystals from the solvent system by filter. Next, the mother liquid was evaporated again under the vacuum at 50 °C to give a residue, which was treated on the basis of aforementioned process until no actein monitored by TLC. Finally, 113 g of actein was obtained.

The mother liquid was evaporated under vacuum at 50 °C, and a residue (40 g) was obtained. Then, the residue was separated by a silica gel column (petroleum ether/MeCO_2_ = 10:1, 2:1, 1:1) to obtain three parts. Fraction (petroleum ether/MeCO_2_ = 1:1, 20 g) was separated by a silica gel column (CHCl_3_/MeCO_2_ 20:1, 10:1, 5:1, 0:1) and yields eight fractions (A.1–A.8). Fraction A.8 (4.6 g) was separated by RP-18 with a gradient elution of MeOH/H_2_O (60:40 to 90:10) to yield four subfractions A8.1–A8.4. Fraction A8.2 (1.7 g) was subjected to a silica gel column (PE/Me_2_CO = 5:1, 2:1, 1:1) into three parts (A8.2.1‒A8.2.3). Fraction A.8.2.1 (300 mg) was treated using RP-18 with a gradient elution of MeOH/H_2_O (60:40 to 90:10) to yield compound **1** (10 mg), compound **7** (5 mg), compound **8** (9 mg). Fraction A8.2.2 (700 mg) was divided by RP-18 with a gradient elution of MeOH/H_2_O (60:40 to 90:10) to yield compound **2** (9 mg), compound **4** (5 mg), compound **5** (5 mg). Fraction A.8.2.3 (200 mg) was separated by RP-18 with a gradient elution of MeOH/H_2_O (60:40 to 90:10) to yield compounds **3** (6 mg) and **6** (11 mg).

### Characteristic Data of Compounds

Acteol-3-*O*-[2′-*O*-(*E*)-2-butenoyl]-*β*-d-xylopyranoside (**1**): white, amorphous powder; [*α*]_D_^22^ = − 86.9 (*c* 0.86, MeOH); UV (MeOH) *λ*_max_ (log *ε*) 197.4 (4.05) nm; IR (KBr) *ν*_max_; 3436, 3038, 2728, 1734, 1652, 1457, 1381, 1043, 985 cm^−1^; ^1^H and ^13^C NMR data see Tables 1 and 2; HRESIMS *m/z* 743.4011 [M−H]^−^ (calcd C_41_H_59_O_12_, 743.4012).

25-*O*-acetylcimigenol-3-*O*-[2′-*O*-3-methoxy-3-oxo-propionyl]-*β*-d-xylopyranoside (**2**): white, amorphous powder; [*α*]_D_^22^ = − 22.6 (*c* 0.21, MeOH); UV(MeOH) *λ*_max_(log *ε*) 253.4 (3.05), 196.4 (3.60) nm; IR (KBr) *ν*_max_; 3436, 3034, 2727, 1739, 1638, 1457, 1368,1308, 1070, 980 cm^−1^;^1^H and ^13^C NMR data see Tables 1 and 2; HRESIMS *m/z* 761.4119 [M−H]^−^ (calcd C_41_H_61_O_13_, 761.4118).

25-*O*-acetylcimigenol-3-*O*-[3′-*O*-3-methoxy-3-oxo-propionyl]-*β*-d-xylopyranoside (**3**): white, amorphous powder; [*α*]_D_^22^ = − 31.0 (*c* 0.57, MeOH); UV(MeOH) *λ*_max_(log *ε*) 195.8 (3.62) nm; IR (KBr) *ν*_max_; 3467, 3034, 2870, 1737, 1631, 1547, 1475, 1368, 1338, 1037, 992, 865 cm^−1^;^1^H and ^13^C NMR data see Tables 1 and 2; HRESIMS *m/z* 761.4115 [M−H]^−^ (calcd C_41_H_61_O_13_, 761.4118).

25-*O*-methoxycimigenol-3-*O*-[4′-*O*- acetyl]-*β*-d-xylopyranoside (**4**): white, amorphous powder; [*α*]_D_^22^ = − 52.7 (*c* 1.54, MeOH); UV(MeOH) *λ*_max_(log *ε*) 195.6 (3.61) nm; IR (KBr) νmax; 3452, 3035, 2869, 1741, 1631, 1458, 1367, 1308, 1051, 993, 865 cm^−1^; ^1^H and ^13^C NMR data see Tables 1 and 2; ESIMS *m/z* 721.4165 [M+HCOO]^−^ (calcd C_39_H_61_O_12,_ 721.4169).

### Cytotoxicity Assay

A panel of human tumor cell lines was used: promyelocytic leukemia HL-60, hepatocellular carcinoma SMMC-7721 and HepG2, alveolar basal epithelial carcinoma A-549, estrogen receptor positive breast carcinoma MCF-7, primary colon carcinoma SW-480 and myelogenous leukemia K-562. The cells lines were obtained from Shanghai Cell Bank of China. All cells were cultured in RPMI-1640 or Dulbecco’s modified Eagle’s medium (Hyclone, U.S.A.), supplemented with 10% fetal bovine serum (Hyclone) at 37 °C in a humidified atmosphere with 5% CO_2_. Growth inhibition by compounds **1**–**4** on HL-60, SMMC-7721, A-549, MCF-7 and SW-480 cells was tested by 3-(4,5-dimethylthiazol-2-yl)-2, 5diphenyltetrazolium bromide (MTT) assay [24].

### Hydrolysis and Identification of the Sugar Moieties in Compounds **1**–**4**

The new compounds **1**‒**4** (3 mg of each) were separately dissolved in MeOH (5 ml); 4% K_2_CO_3_ (5 ml) was added, and the solution was stirred at room temperature overnight. The solution was neutralized with 10% HOAc and extracted with EtOAc (3 × 10 ml). After removal of the solvent, the EtOAc extract was dissolved in MeOH (5 ml) and refluxed with 0.5 N HCl (1 ml) for 4 h. Each reaction mixture was diluted with H_2_O and extracted with CHCl_3_. The water layer was applied on an Amberlite IR-35 (5 ml) column, and the resultant fraction was concentrated in *vacuo* to give a monosaccharide, which had an R_*f*_ 0.4 (EtOAc–CHCl_3_‒MeOH‒H_2_O, 3:2:2:1) and specific rotation{[R]25 D +21.22 (*c* 0.10, H_2_O)} comparable to those of d-xylose (*Sigma*-*Aldrich*).

## Electronic supplementary material

Below is the link to the electronic supplementary material.

Supplementary material 1 (DOCX 11586 kb)
