# Supplementary material for: Cytotoxic Cycloartane Triterpenoid Saponins from the Rhizomes of Cimicifuga foetida
Source: Nat Prod Bioprospect. 2019 Jun 18;9(4):303–10. doi: 10.1007/s13659-019-0214-1 (PMC6646490; doi:10.1007/s13659-019-0214-1)

Supporting Information Available

**Five New Cycloartane Triterpenoids from *Cimicifuga foetida.***

Jing Lu^a,b^, Xing-Rong Peng ^a^, Da-Shan Li ^a^, Qiang-Qiang Shi ^a,b^, Ming-hua Qiu^a,b,^*

^a^State Key Laboratory of Phytochemistry and Plant Resources in West China, Kunming Institute of Botany, Chinese Academy of Sciences, Kunming 650201, China

^b^University of Chinese Academy of Sciences, Beijing 100049, China

**Correspondence**

***MingHua Qiu***

State Key Laboratory of Phytochemistry and Plant Resources in West China

Kunming Institute of Botany, Chinese Academy of Sciences

132 LanHei Road

Kunming 650201, Yunnan, P. R. China.

Tel: +86-871-65223327

Fax: +86-871-65223255

Email address: [mhchiu@mail.kib.ac.cn](mailto:mhchiu@mail.kib.ac.cn)

**Supporting Information List**

Characterization Data of New Compounds

**S 1.** ^1^H NMR spectrum of compound **1** (C_5_D_5_N, 600 MHz).

**S 2.** ^13^C NMR spectrum of compound **1** (C_5_D_5_N, 150 MHz).

**S 3.** HMBC spectrum of compound **1**.

**S 4.** ^1^H-^1^H COSY spectrum of compound **1**.

**S 5.** HSQC spectrum of compound **1**.

**S 6.** ROESY spectrum of compound **1**.

**S 7.** HR-ESIMS spectrum of compound **1**

**S 8.** IR spectrum of compound **1**

**S 9.** ^1^H NMR spectrum of compound **2** (C_5_D_5_N, 600 MHz).

**S 10.** ^13^C NMR spectrum of compound **2** (C_5_D_5_N, 150 MHz).

**S 11.** HMBC spectrum of compound **2**.

**S 12.** ^1^H-^1^H COSY spectrum of compound **2**.

**S 13.** HSQC spectrum of compound **2**.

**S 14.** ROESY spectrum of compound **2**.

**S 15.** HR-ESIMS spectrum of compound **2.**

**S 16.** IR spectrum of compound **2.**

**S 17.** ^1^H NMR spectrum of compound **3** (C_5_D_5_N, 600 MHz).

**S 18.** ^13^C NMR spectrum of compound **3** (C_5_D_5_N, 150 MHz).

**S 19.** HMBC spectrum of compound **3**.

**S 20.** ^1^H-^1^H COSY spectrum of compound **3**.

**S 21.** HSQC spectrum of compound **3**.

**S 22.** ROESY spectrum of compound **3**.

**S 23.** HR-ESIMS spectrum of compound **3.**

**S 24.** IR spectrum of compound **3.**

**S 25.** ^1^H NMR spectrum of compound **4** (C_5_D_5_N, 600 MHz).

**S 26.** ^13^C NMR spectrum of compound **4** (C_5_D_5_N, 150 MHz).

**S 27.** HMBC spectrum of compound **4**.

**S 28.** ^1^H-^1^H COSY spectrum of compound **4**.

**S 29.** HSQC spectrum of compound **4**.

**S 30.** ROESY spectrum of compound **4**.

**S 31.** HR-ESIMS spectrum of compound **4.**

**S 32.** IR spectrum of compound **4.**

**S 33.** speciﬁc rotation of D-xylopyranoside

S1. ^1^H NMR spectrum (pyridine-*d*_5_, 600MHz) of compound **1**

**S 2.** ^13^C NMR spectrum of compound **1** (C_5_D_5_N, 150 MHz).

**S 3.** HMBC spectrum of compound **1**.

**S 4.** ^1^H-^1^H COSY spectrum of compound **1**.

**S 5.** HSQC spectrum of compound **1**.

**S 6.** ROESY spectrum of compound **1**.

**S 7.** HR-ESIMS spectrum of compound **1**


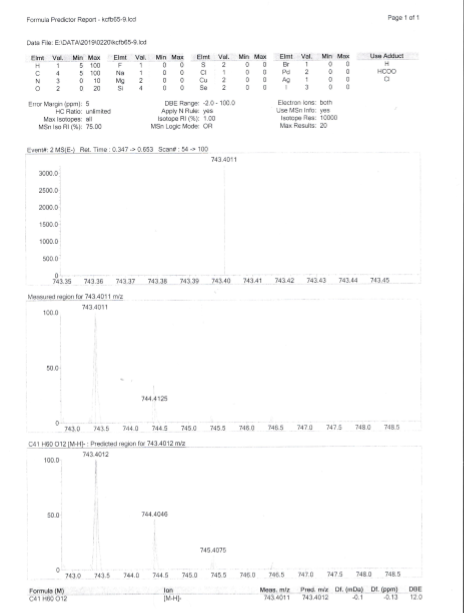


**S 8.** IR spectrum of compound **1**


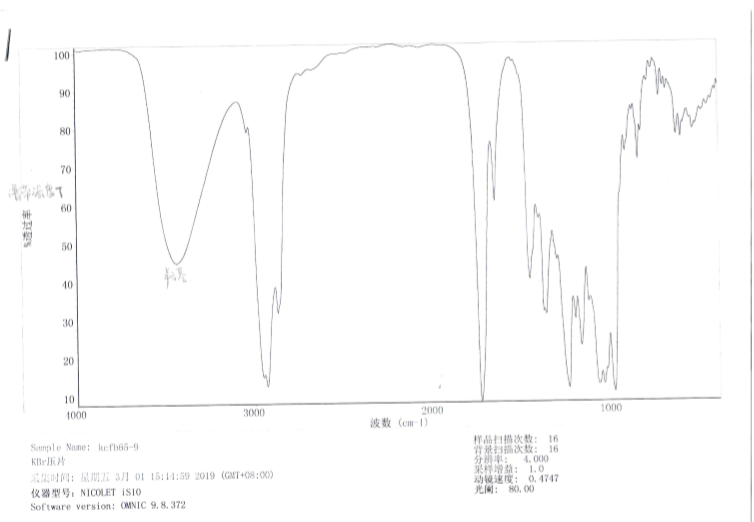


**S 9. ^1^H** NMR spectrum of compound **2** (C_5_D_5_N, 150 MHz).

**S 10.** ^13^C NMR spectrum of compound **2** (C_5_D_5_N, 150 MHz).

**S 11.** HMBC spectrum of compound **2**.

**
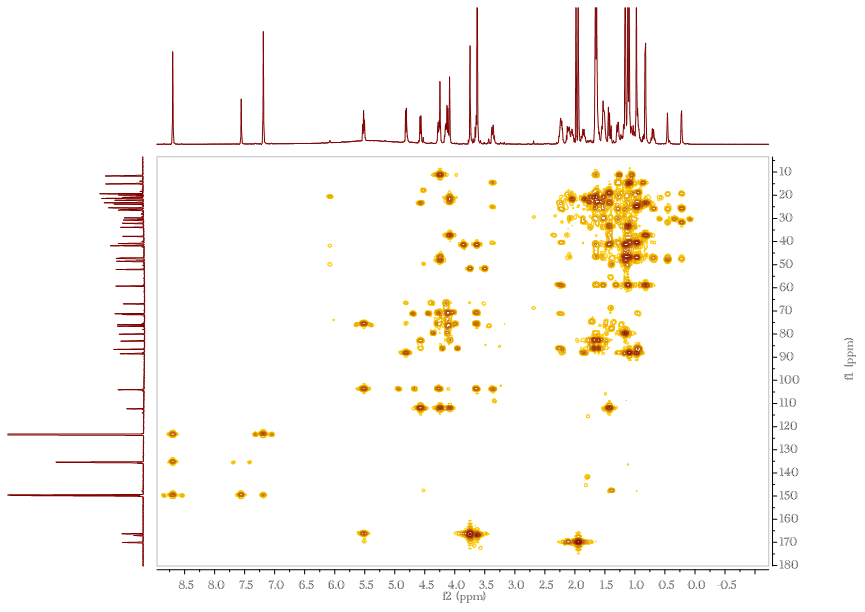
**

**S 12.** ^1^H-^1^H COSY spectrum of compound **2**.

**S 13.** HSQC spectrum of compound **2**.

**S 14.** ROESY spectrum of compound **2**.

**S 15.** HR-ESIMS spectrum of compound **2.**


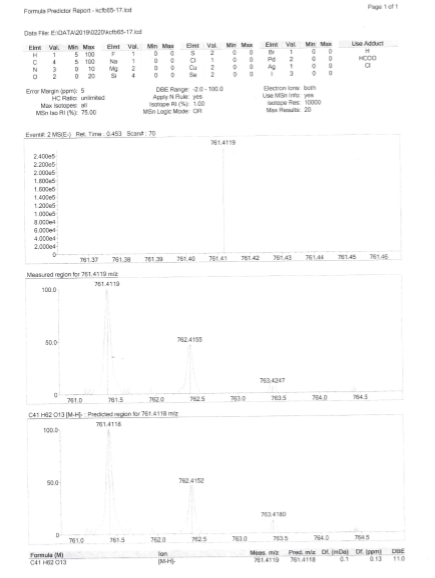


**S 16.** IR spectrum of compound **2.**


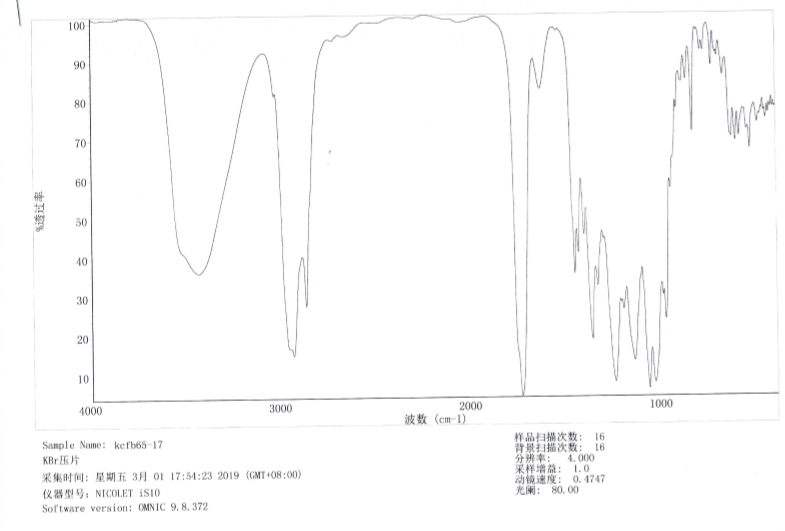


**S 17.** ^1^H NMR spectrum of compound **3** (C_5_D_5_N, 600 MHz).

**S 18.** ^13^C NMR spectrum of compound **3** (C_5_D_5_N, 150 MHz).

**S 19.** HMBC spectrum of compound **3**.

**S 20.** ^1^H-^1^H COSY spectrum of compound **3**.

**S 21.** HSQC spectrum of compound **3**.

**S 22.** ROESY spectrum of compound **3**.

**S 23.** HR-ESIMS spectrum of compound **3.**


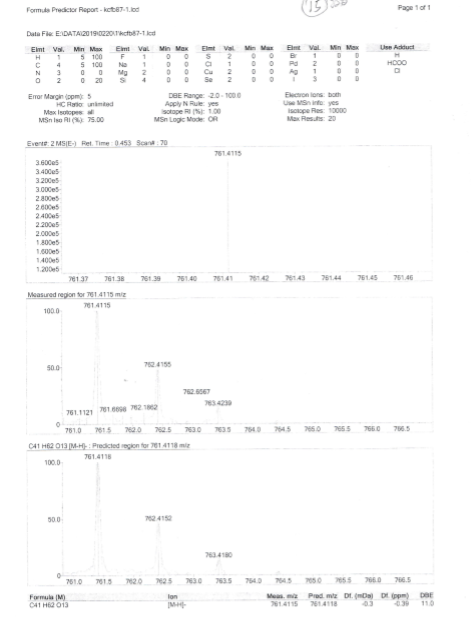


**S 24.** IR spectrum of compound **3.**


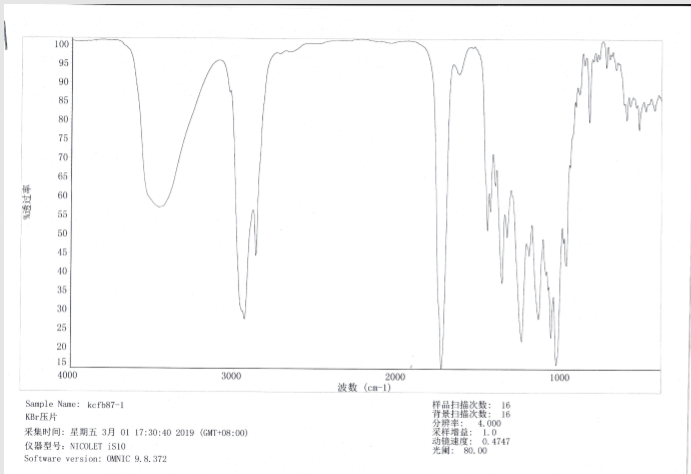


**S 25.** ^1^H NMR spectrum of compound **4** (C_5_D_5_N, 600 MHz).

**S 26.** ^13^C NMR spectrum of compound **4** (C_5_D_5_N, 150 MHz).

**S 27.** HMBC spectrum of compound **4**.

**S 28.** ^1^H-^1^H COSY spectrum of compound **4**.

**S 29.** HSQC spectrum of compound **4**.

**S 30.** ROESY spectrum of compound **4**.

**S 31.** HR-ESIMS spectrum of compound **4.**


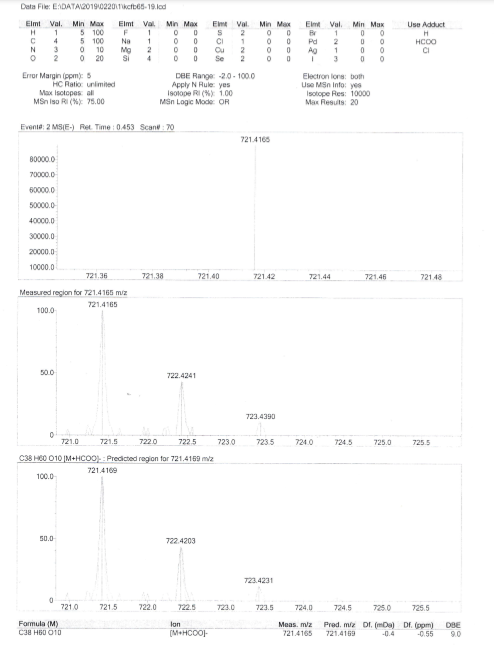


**S 32.** IR spectrum of compound **4.**


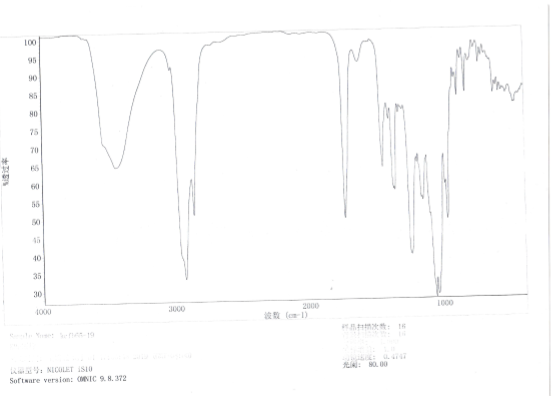


**S 33.** Speciﬁc rotation of D-xylopyranoside


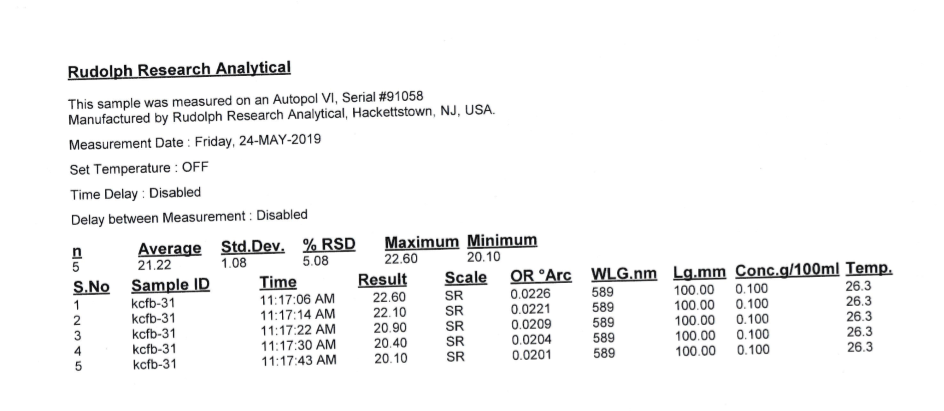

Supplement: Supplementary file 1 — Supplementary material 1 (DOCX 11586 kb) [file 13659_2019_214_MOESM1_ESM.docx]
